# Type 0 Spinal Muscular Atrophy in rare association with congenital Contracture and generalized osteopenia

**Published:** 2018

**Authors:** Aditi SINGH, Poonam DALAL, Jasbir SINGH, Pooja TRIPATHI

**Affiliations:** 1 Department of Pediatrics, PGIMS, Rohtak, Haryana, India.

**Keywords:** Spinal muscular atrophy, Type 0, Hypotonia, osteopenia

## Abstract

Spinal muscular atrophy (SMAs) is a group of rare autosomal recessive diseases in which there is degeneration of alpha motor neurons in the spinal cord leading to progressive distal motor weakness. Here we report a case of type 0 SMA in a female neonate born at the Department of Pediatrics, PGIMS, Rohtak (Haryana) India, associated with generalized osteopenia and bony deformity in form of unilateral club foot. It may be emphasized that diagnosis of SMA should be kept in mind as a differential in cases of unexplained severe generalized hypotonia and severe respiratory compromise immediately after birth.

## Introduction

Spinal muscular atrophy (SMA) is a genetic disorder involving nervous system presenting with progressive distal motor neuron weakness. Underlying degeneration of alpha motor neurons in the spinal cord results in progressive symmetrical proximal muscle weakness and paralysis. This disease is caused by homozygous mutation of survival motor neuron (SMN)-1 gene, generally showing the absence of SMN 1 axon 7. A new variant of SMA called type 0 which had intrauterine onset and presents with hypotonia with a fatal and progressive course had been reported (1-3). An association of SMA with generalized osteopenia and congenital contracture with Ubiquitin-activating enzyme 1 (UBE-1) mutation is reported (4).

## Case Report

A female neonate was born to second gravida mother from nonconsanguineous marriage by vertex vaginal delivery at 37 weeks gestation. The neonate did not cry immediately after birth and required intensive resuscitation at birth. The baby never had spontaneous respiratory effort and required mechanical ventilation immediately after birth. Extreme hypotonia of all limb and truncal muscles was noted with a strikingly alert look. There was no spontaneous movement of limbs. Deep tendon reflexes were absent. Baby responded to tactile stimuli. Extraocular and facial muscles were spared. There was unilateral club foot on examination. Systemic examination revealed no other abnormality.

**Figure 1:**
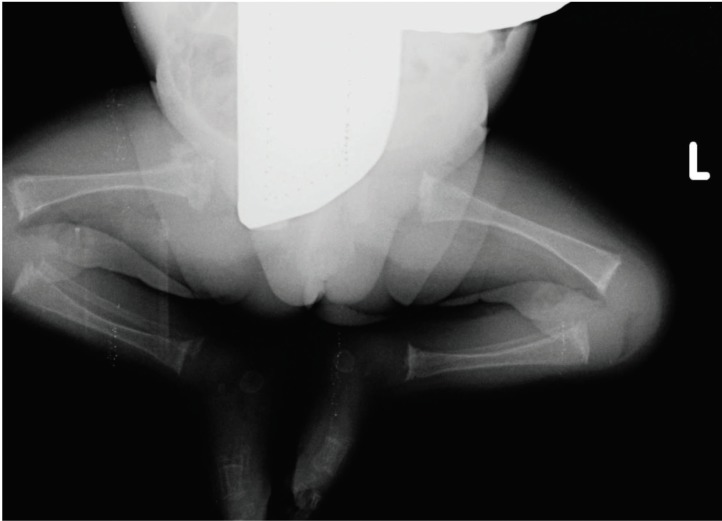
X-Ray (bilateral lower limbs) showing generalized osteopenia.

Antenatal history and records were suggestive of polyhydramnios yet with decreased fetal movement from 32 weeks of gestation. Apart from that antenatal period was uneventful without signs and symptoms of other medical ailments. There was a history of the death of a sibling in neonatal period due to the unidentified cause. 

Baby required sustained mechanical ventilation; seizures were noted on the second day of life and were controlled with phenobarbitone. The alert look was in contrast to marked hypotonia and absence of spontaneous muscle activity. The infant continued to be ventilator dependent throughout the hospital stay. She was noticed to have generalized osteopenia on X-ray (Fig. 1). 

In view of marked hypotonia and history of the death of a sibling in the neonatal period, gene analysis for SMN was done and showed homozygous mutation of exon 7 consistent with the diagnosis of Spinal Muscular Atrophy. The baby succumbed to severe pneumonia and died after fifteen days of birth. The parents were called in follow up for genetic counseling in view of recurrence risk of the SMA.

## Discussion

 Spinal Muscular Atrophy (SMA) is a disorder of anterior horn cells of the spinal cord and follow the autosomal recessive pattern of inheritance. It manifests as progressive symmetrical weakness and proximal muscles atrophy (1). The reported incidence varies from 1 in 6,000 to 1 in 10,000 live births with the carrier frequency of 1/40-1/60. Clinically four group of the disease had been identified affected according to the age of onset and progression of weakness. Children with type I SMA are the most severely affected ones and they usually have symptoms before six months of age. These patients are unable to sit and usually die within 1-2 yr as a result of respiratory insufﬁciency and infections. Type II patients have a usually milder presentation and the patient may survive up to adolescence age. However, they are unable to stand without support. Type III SMA is said to be the mildest form of all SMAs (1). These three types are allelic and the majority are caused by homozygous deletions of the Survival Motor Neuron (SMN) gene localized on chromosome region 5q13 (2). In addition to these classical SMA types, unusual SMA variants have been described (3-5). A new form of SMA (type 0) had been reported (1). This variant has fatal course with intrauterine onset, leading to profound hypotonia, facial weakness, and may lead to death within the ﬁrst three months1. Homozygous mutations of the survival motor neuron 1 (SMN1) gene are thought to be the underlying cause. The available diagnostic test by showing the absence of SMN1 exon 7 demonstrates the homozygous deletion of the SMN1 gene. These genetic tests may have sensitivity 95% and nearly 100% specificity. 

Type 0 SMA has recently been documented and present with reduced muscle movement in utero, profound hypotonia, severe asphyxia, respiratory insufficiency at birth and need for resuscitation and mechanical ventilatory support (1). An alert look in sharp contrast to severe hypotonia has been uniformly observed. The index case too had an antenatal onset with decreased fetal movement from 32 weeks of gestation, severe asphyxia needing resuscitation and mechanical ventilation, profound hypotonia and a striking alert look. Like other cases reported till date the index case was also ventilator dependent and died in the neonatal period. 

Seizures in case of SMA type 0 are generally described as consequences of hypoxic-ischemic encephalopathy, secondary to birth asphyxia. In the reported case, too, neonate had seizures due to hypoxic-ischemic encephalopathy. There are several reports of SMA with additional feature including long bone fracture, osteopenia, diaphragmatic paralysis, congenital heart disease, congenital contracture (6, 7). Spinal muscular atrophy presenting with generalized osteopenia and congenital contracture have been described in association with Ubiquitin-activating enzyme (1) (UBE1) mutation in literature (4). The index case also had generalized osteopenia and unilateral club foot. The consistent radiographic findings in all cases of the neuromuscular disease in the study by Rodriguez JI showed multiple diaphyseal or metaphyseal fractures or both, primarily involving the long bones of the upper extremities (7). Treatment is mainly supportive as there is no definitive cure. Chest physiotherapy, antibiotics form the important part of improving quality of life of patients. Sodium valproate and hydroxyurea are used but their efficacy is unproven. Various trials are under process to find out the role of gene therapy and stem cell therapy to improve the motor neuron protein life. 

Since genetic basis of SMA had been described and as there is a recurrence risk of 25%, prenatal diagnosis may be of help for couples who previously had a child affected with SMA. The role of follow-up coordination has to be managed by an expert in neuromuscular disorders who is able to plan a multidisciplinary intervention that includes pulmonary, gastroenterology and nutritional management.

**In conclusion, **SMAs are fatal neuromuscular disorders with genetic predisposition. Prenatal diagnosis and selective termination of pregnancy is an acceptable preventive option due to absence of definitive treatment. With recent advances in molecular and genetic interventions there may be development of effective drugs and therapeutic interventions for spinal muscular atrophy in future. 

## Conflict of Interest

The authors declare that there is no conflict of interests.
